# PetBERT: automated ICD-11 syndromic disease coding for outbreak detection in first opinion veterinary electronic health records

**DOI:** 10.1038/s41598-023-45155-7

**Published:** 2023-10-21

**Authors:** Sean Farrell, Charlotte Appleton, Peter-John Mäntylä Noble, Noura Al Moubayed

**Affiliations:** 1 Department of Computer Science, Durham University, Durham, UK; 2 Centre for Health Informatics, Computing, and Statistics, Lancaster Medical School, Lancaster University, Lancaster, UK; 3 Institute of Infection, Veterinary and Ecological Sciences, University of Liverpool, Liverpool, UK; 4 Evergreen Life Ltd, Manchester, UK

**Keywords:** Data mining, Machine learning

## Abstract

Effective public health surveillance requires consistent monitoring of disease signals such that researchers and decision-makers can react dynamically to changes in disease occurrence. However, whilst surveillance initiatives exist in production animal veterinary medicine, comparable frameworks for companion animals are lacking. First-opinion veterinary electronic health records (EHRs) have the potential to reveal disease signals and often represent the initial reporting of clinical syndromes in animals presenting for medical attention, highlighting their possible significance in early disease detection. Yet despite their availability, there are limitations surrounding their free text-based nature, inhibiting the ability for national-level mortality and morbidity statistics to occur. This paper presents PetBERT, a large language model trained on over 500 million words from 5.1 million EHRs across the UK. PetBERT-ICD is the additional training of PetBERT as a multi-label classifier for the automated coding of veterinary clinical EHRs with the *International Classification of Disease 11* framework, achieving F1 scores exceeding 83% across 20 disease codings with minimal annotations. PetBERT-ICD effectively identifies disease outbreaks, outperforming current clinician-assigned point-of-care labelling strategies up to 3 weeks earlier. The potential for PetBERT-ICD to enhance disease surveillance in veterinary medicine represents a promising avenue for advancing animal health and improving public health outcomes.

## Introduction

Disease surveillance in animals aims to provide decision-makers with real-time spatial and temporal insights into the prevalence of specific diseases within the animal population. Such information facilitates prompt detection of outbreaks or the emergence of new diseases (e.g. new pathogen or environmental factor causing disease) and allows prompt measures to limit their effect. Several diseases affecting companion animals have zoonotic potential, including Brucella^1^, Pasteurella^2^, and leptospirosis^3^, highlighting the critical need for vigilance. Additionally, emerging diseases may affect companion animals, including pets living in close proximity to humans^4^. Electronic health record (EHR) surveillance data collected from veterinary clinics can provide a snapshot of the current health of the animal population. Surveillance schemes such as the Small Animal Veterinary Surveillance Network (SAVSNET) collect and curate EHRs in near real-time on a National scale (UK)^5^. However, scaling up the utility of first-opinion veterinary EHRs presents challenges, as veterinary practices often lack dedicated disease coding personnel. Furthermore, coding standards, though in existence, are inconsistently applied due to time constraints, resulting in limited structured data. Naming conventions and clinical narrative structures are practice-specific, often without harmonised recording of clinical features. Hence, there is a need to develop and implement practical solutions to overcome these challenges and enhance the quality and usability of veterinary EHRs to benefit animal and human health.

*The International Classification of Diseases (ICD)* framework has long been considered the gold standard for disease coding within human medicine, categorising diseases, disorders and injuries into a hierarchical, numerical structure^6^. ICD contributes significantly to advancing human epidemiology by establishing a universal framework for comparing mortality and morbidity data, transcending language barriers, and simplifying the analysis of health-related information^7^. Integrating the ICD framework into veterinary medicine, with a specific focus on monitoring the companion animal population for variations in incidence of ICD classifications, offers a promising avenue for the early detection of emerging zoonotic diseases. This could facilitate timely intervention and set the stage for establishing essential coding standards within this unique context^8^. Nevertheless, there needs to be more development in structured disease coding within veterinary medicine^8^. The unstructured format of veterinary clinical narrative notes presents significant challenges in manual annotation. These challenges stem from the time demands placed on annotators and the heightened risk of increased annotation errors, particularly when dealing with extensive datasets^9,10^.

Recent Natural Language Processing (NLP) advances have yielded substantial enhancements across diverse text-driven tasks. A critical development in this field has been the transformer architecture incorporating the novel self-attention mechanism^11^. This architecture was first implemented in the Bidirectional Encoder Representation for Transformers (BERT)^12^ ushering in a new era of state-of-the-art performance across various NLP benchmarks^13,14^. Transformers have consistently held their position as the leading architecture and serve as the foundation for many of the newer and considerably larger language models, including LaMDA^15^, GPT-3^16^, BLOOM^17^, and OPT^18^. Performance improvements are observed for domain-specific language tasks when a transformer model trained on a generalised corpus is fine-tuned on a domain-specific corpus, proposed as the Universal Language Model Fine-Tuning (ULMFiT) framework^19^. Notable examples include BioBERT, which underwent additional training across 4.5b words from PubMed abstracts and 13.5B words from PMC full-text articles and BioClinicalBERT across 2 million of the MIMIC-III EHR dataset, both sporting significantly improved performances across a degree of domain-specific tasks such as MedNLI and i2b2 compared to the original BERT^20–25^

More recently, recurrent neural networks have been employed, such as the 2017 study utilizing Long short-term memory (LSTM)^26^ and convolutional neural networks^27,28^. Transformers have played a pivotal role in pushing the boundaries of this field, ultimately attaining new state-of-the-art performances^29,30^. Nevertheless, attaining an accurate end-to-end automated coding system remains a significant challenge. The current state-of-the-art MIMIC-III discharge summary ICD-9 classification task is at an F1 score of 60%^31,32^. The difficulty in achieving this goal reflects the variation in structure, notation, length and completeness of clinical records, which hampers the development of a model to create meaningful links between natural language and the knowledge representation of the ICD framework^33^. In the domain of veterinary healthcare, the prior contributions of Nie et al. and Zhang et al. introduced DeepTag and VetTag, respectively^34,35^. These methodologies established automated frameworks for SNOMED-CT coding labels, leveraging an extensive compilation of referral clinic notes annotated by attending clinicians. Nevertheless, primary veterinary care often lacks equivalent diagnostic certainty to referral clinics, frequently drawing upon specialised expertise and a wealth of supplementary diagnostic information. First-opinion EHRs will, therefore, often have a listing of disease differentials and symptoms instead. This paper distinguishes itself from earlier works by effectively addressing the complexities and uncertainties inherent within the primary care context. Given the continuous daily influx of EHRs into our dataset and the intricate nature of models required for accurate record annotation, the task presents an ideal opportunity for applying a large language model.

An automated system for annotating EHR with ICD-11 classes could support monitoring disease outbreaks in the UK population of companion animals and serve as a public health warning for the early detection of potential zoonotic disease. A WHO report for emerging diseases estimates 60% of all human diseases currently understood and 75% of emerging diseases with a human impact between 1980 and 2010 have been of zoonotic origin^36^. This has since yielded the One Health initiative, a strategy devised by the WHO aimed to unify human, animal and environmental health with a specific focus on controlling zoonoses^37^. Despite this, surveillance networks are primarily developed for understanding disease epidemiology within humans and ultimately omit an unrealised data source that could allow for earlier disease detection if such systems were developed for the companion animal population. Monitoring for companion animal national disease outbreaks can inform clinicians of what symptoms to look out for, researchers to determine the exact aetiological agent, and form an automated reporting tool for public health agencies to allow rapid notification of changes in disease activity. However, this proves challenging where the exact symptoms for what to look for may be unknown in the case of a novel disease or syndrome and where the importance of such outbreak reduction strategies relies on realising such an outbreak has occurred with as few cases as possible.

Previous works by the SAVSNET group revealed a known severe disease outbreak from December 2019 to March 2020 presenting as severe vomiting in dogs associated with the canine enteric coronavirus^38,39^. Gastrointestinal disease, in particular, can have a high financial burden to manage, has a high impact on animal welfare and, depending on aetiology, may reflect disease transmissible to animals and humans^40,41^. However, despite this, a coordinated population-level approach to detecting gastrointestinal outbreaks within companion animals is still lacking. Currently, practitioners participating in SAVSNET append a ‘main presenting complaints’ (MPC) label at the end of every consultation, selecting from one of ten labels; these are ‘gastroenteric’, ‘kidney disease’, ‘post operative’, ‘pruritus’, ‘respiratory’, ‘trauma’, tumour’, ‘vaccination’, ‘other healthy’ and ‘other unwell’. These labels have assisted the research of broad disease themes; however, they are highly susceptible to errors for the same reasons stated above with human coding. The ‘gastroenteric’ MPC data exposed the gastrointestinal outbreak, but this method only permits one label per record and thus fails to capture multisystemic diseases. We suggest a multi-label classification system that leverages the high-order ICD-11 framework to overcome human annotation errors and enable more diverse and accurate labels over the current MPC system. This system more than doubles the number of detection tracks and enhances the surveillance of future outbreaks.

Here we introduce ‘PetBERT,’ a robust clinical language model based on BERT-base, that underwent extensive training on a corpus exceeding 500 million tokens extracted from first-opinion clinical free-text EHRs across the UK. PetBERT was then applied to the downstream task of multi-label classification of the ICD-11 framework to become PetBERT-ICD. PetBERT has been exposed to diverse linguistic patterns and domain-specific knowledge, enhancing its reliability and adaptability in the analysis of clinical notes. Our study leverages a large and diverse dataset of over 8 million electronic health records collected from 253 practices. To ensure confidence in the generalisability of our model to clinical notes written by any first-opinion clinician, we partitioned our training and test sets at a practice level, aiming to ensure that the clinical notes utilized for testing remained distinct from those contributed to the training sets. We contribute a methodology to attain high classification accuracy on multi-label models from relatively small training sets using the adapted teacher-student framework proposed by Yalniz et al. through the iterative training of smaller, binary classification models for each of the ICD-11 labels before applying these to a unified dataset which was used to train and evaluate the final multi-label model^42^. Lastly, we illustrate the effectiveness of PetBERT-ICD through a case study in which it successfully identifies disease outbreaks emerging within the UK companion animal population, outperforming currently deployed strategies.

## Results

This section details the processing and segmentation methods applied to the initial datasets, aiming to enhance the generalisability of our models. We explore the additional fine-tuning of PetBERT and the downstream classification tasks to produce PetBERT-ICD for the automated ICD-11 classification tool. To underscore the real-world applicability of our approach, we demonstrate its effectiveness in disease outbreak detection with a comparison to the current clinician assigned at point-of-care label methodology.

### Data extraction

The SAVSNET dataset comprises 8,010,544 EHRs collected between April 2014 and October 2022. Records with missing data points, such as age (n = 35,600), owner’s postcode (n = 77,250), sex (n = 2869), and breed (n = 375,981), were excluded. After retaining only records for dogs (n = 5,275,843) and cats (n = 2,062,074), 7,333,384 records remained. At this stage, records were split based on their respective veterinary practice such that of the 253 practices, 177 became the training set and the remaining 76 the test set. This approach to randomly select records based on practice rather than by random selection is in light of a recent review determining that algorithms trained on institution-level health records were not generalisable to other institutions^43^. No additional prepossessing was done to the text. The test set records were set aside for later use as the inference set in the model’s application for outbreak analysis. The remaining 5,171,999 records were utilised for further pretraining of the PetBERT model. For the ICD-11 dataset, 7500 records were randomly selected and annotated, with the specific case definition for each ICD-11 being “an animal with symptoms or a diagnosis corresponding to the specific ICD-11 code”. These records were then separated into 20 independent data sets. Named diseases were categorised based on their appearance within the ICD-11 framework, with any veterinary-specific conditions discussed with a practising clinician.

A practising veterinary clinician extracted a random subset of 1000 records from the test set for manual annotation to establish the model’s final performance metrics. We conducted reviewer agreement checks to ensure the MPC labels’ accuracy and consistency. In cases of disagreement, a ‘corrected’ label was proposed. Among the 1000 records reviewed, a significant portion—396 records (39.6%)—did not agree with the initial labelling. Most disagreements occurred when the attending practitioner had assigned a consultation to the ‘other’ category. However, it was determined upon review that 61.0% of these records could be more accurately reassigned to one of the eight more diagnostic MPC labels. This finding underscores the complexity of accurately categorising clinical records, a challenge also encountered in the realm of human medicine^44,45^. Subsequently, we annotated the dataset using a high-order ICD-11 labelling set comprising 20 syndromic disease categories. This annotation allowed for the flexibility to assign multiple labels to each EHR.

### Model performance

**Figure 1:**
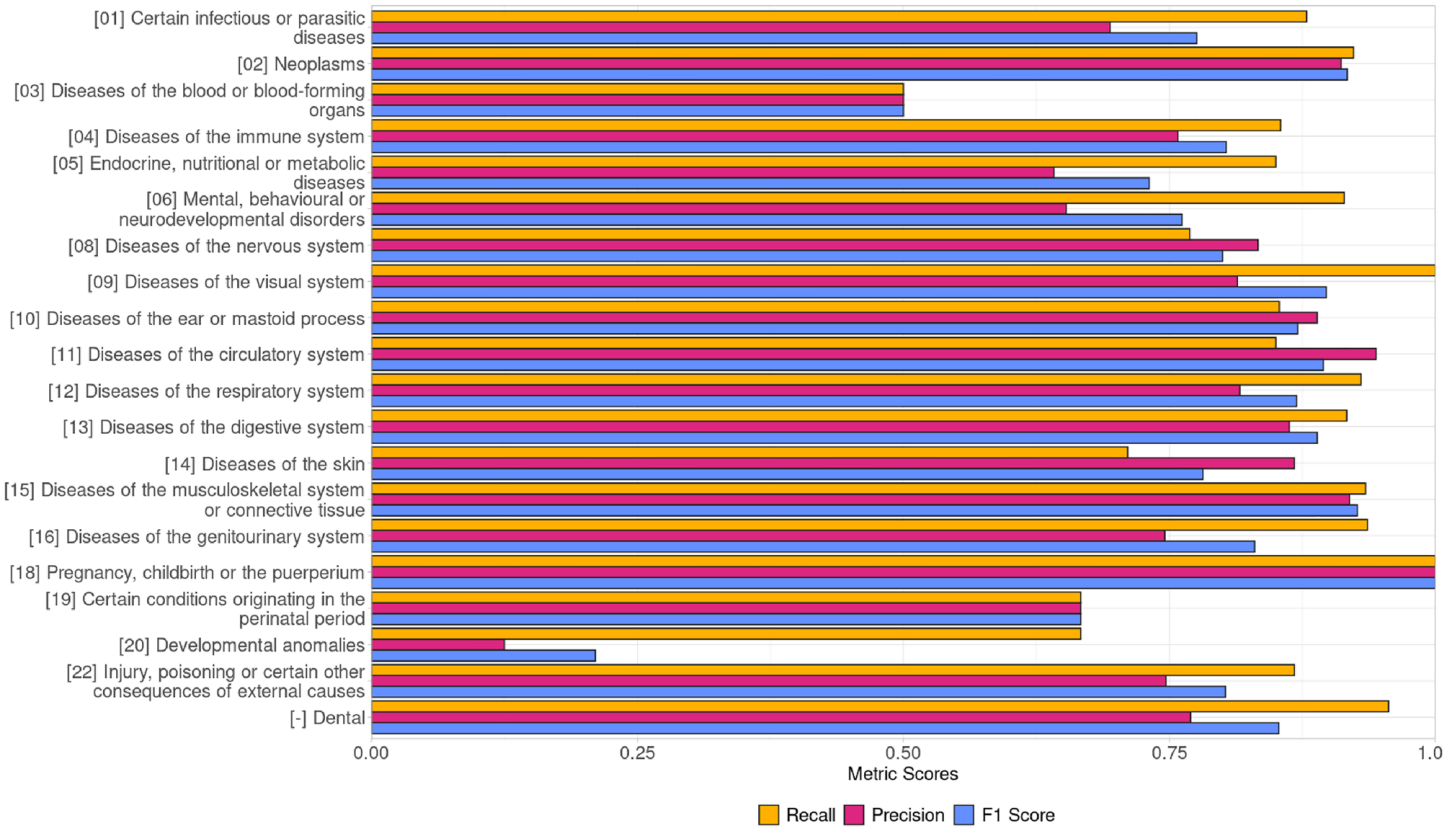
Performance metrics for each of the high level 20 ICD-11 disease coding classes using the PetBERT-ICD model, presented as recall (Yellow, top), precision (Red, middle), and F1 score (Blue, bottom) scores on a clinician-annotated test set of 1000 samples.

For each of the 20 ICD-11 labels, a binary classification model was trained from manually annotated records. 20% of the dataset was removed for the validation set for model training. Table 1 presents a compilation of performance metrics for the individual binary classification models and the final PetBERT-ICD relative to the produced evaluation sets, including their respective number of in-class examples used within the training. The 20 binary classification models were then applied to two random samples of 250,000 records, which were pulled from the training dataset such that each record could have up to 20 labels. The first dataset was then used as the training set for PetBERT-ICD, a multi-label classification model with the capacity to select up to 20 labels for a single narrative, and evaluated against the second 250,000 dataset produced. A complete outline of the proposed data pipeline for PetBERT-ICD can be seen in Fig. 3. Finally, PetBERT-ICD was applied to the 2.1 million records, with 1000 records sampled from this final test set for the manual annotations by a practising vet. This yielded an F1 micro average score of 0.83, with a precision of 0.81 and recall of 0.86, respectively. The full per-label metrics and a demonstration of the multi-labelling capacities of PetBERT-ICD through example model outputs onto deidentified clinical records can be seen in Fig. 1 and Table 2, respectively.

**Table 1:** Performance metrics of 20 binary classification models with in-class examples counted as ‘number of cases’. The multi-label classification model was trained and evaluated using datasets derived from the 20 binary classification models.

	Binary classification model (×20)				Multi-label classification model			
	Precision	Recall	F1	Number of cases	Precision	Recall	F1	Number of cases
[01] Certain infectious or parasitic diseases	0.95	0.95	0.95	300	0.98	0.97	0.97	18,261
[02] Neoplasms	0.92	0.92	0.92	500	0.96	0.90	0.93	22,859
[03] Diseases of the blood or blood-forming organs	0.96	0.96	0.96	350	0.98	0.93	0.95	2287
[04] Diseases of the immune system	0.91	0.91	0.91	300	0.97	0.90	0.94	13855
[05] Endocrine, nutritional or metabolic diseases	0.96	0.97	0.97	500	0.97	0.87	0.91	18,628
[06] Mental, behavioural or neurodevelopmental disorders	0.91	0.91	0.91	450	0.95	0.96	0.96	3214
[08] Diseases of the nervous system	0.87	0.87	0.87	200	0.97	0.94	0.95	22,502
[09] Diseases of the visual system	0.98	0.98	0.98	400	0.95	0.97	0.96	6903
[10] Diseases of the ear or mastoid process	0.95	0.95	0.95	300	0.98	0.87	0.92	17,976
[11] Diseases of the circulatory system	0.91	0.91	0.91	150	0.98	0.94	0.96	10,098
[12] Diseases of the respiratory system	0.91	0.91	0.91	150	0.96	0.95	0.95	33,479
[13] Diseases of the digestive system	0.93	0.93	0.93	250	0.97	0.97	0.97	29,324
[14] Diseases of the skin	0.91	0.91	0.91	250	0.97	0.96	0.97	20,275
[15] Diseases of the musculoskeletal system or connective tissue	0.89	0.89	0.89	250	0.94	0.97	0.96	15,246
[16] Diseases of the genitourinary system	0.98	0.98	0.98	500	0.98	0.98	0.98	21,798
[18] Pregnancy, childbirth or the puerperium	0.89	0.89	0.89	125	0.98	0.94	0.96	657
[19] Certain conditions originating in the perinatal period	0.98	0.98	0.98	200	0.99	0.97	0.98	2115
[20] Developmental anomalies	0.97	0.97	0.97	350	0.98	0.95	0.97	13,485
[22] Injury, poisoning or certain other consequences of external causes	0.95	0.95	0.95	300	0.98	0.87	0.92	23,610
Dental	0.98	0.98	0.98	400	0.99	0.70	0.82	12,698

**Table 2:** Example clinical narratives from PetBERT-ICD multi label classifier outputs with the number of labels ranging from zero to multiple selected for a single given narrative.

Example narrative	Model output
“owner no concerns. defecating, urinating, drinking, eating. no vomiting/diarrohea. clinical exam: nothing abnormal detected. Given tricat and felv vac cbooster”	
“Mammary mass grown ++ − o does not want surgery so try pain relief and monitoring”	[02] Neoplasms
. mass betwenn blade infested,possible cyst/wart.....ab given and remove if given too much problem. heart murmur increasing from grade 2 to grade 4/6..advice to start vetmedin (have a syncope few days ago). check \in 1 month. Next appointment in 1 month	[02] Neoplasm [11] Diseases of the circulatory system
“History: diar over weekend but also lots of urine passed in house. Clinical Exam: bright, eating, no sign of dehydration although dif to examine mouth, temp 101.2, tender abd, no blood on therm. Differential Diagnosis: ? dietary indiscretion +/− UTI. Plan:req urine sample, start on abs and yumpro, hills id. Owners on hols -pay on return”	[01] Certain infectious or parasitic diseases [13] Diseases of the digestive system [16] Diseases of the genitourinary system
“Doing well at this time, slight bradycardia but chest clear and OR fit and healthy managing nicely with deafness. uptodate with worming etc. adv re dental - tartar ++.	[11] Diseases of the circulatory system [10] Diseases of the ear or mastoid process [23] Dental
“O brought in. noticed R ear is quite inflammed and has been scratching at. has had ear infection in the past. seizure described as above. Cx: mucous membranes pink and moist. capillary refill time lt;2. teeth ok. thoracic ausc NAD. abdo palp NAD. L ear fine on otoscopy. yellow/brown dc on otoscopy, slightly inflammed. would not tolerate full otoscopic exam. mentation normal. no propioceptive defecits. O thinks may have inner ear infection given had seizure. advised certainly has OE, cannot rule out as unable to visualise TM but feel inner ear infection unlikely given no head tilt/ataxic gate/circling etc. advised no drops in stock that are safe to treat inner ear infection but as feel unlikely can have trial course of surolan. if vestibular signs develop then stop treatment and owner to ring. discussed seizure. dispensed diazepam and owner to ring if any more seizures”	[01] Certain infectious or parasitic diseases [08] Diseases of the nervous system [10] Diseases of the ear or mastoid process
“cellulitis left fore. very similar to visit on 13th Sept. owner reports appeared to recover well from that then the sting right fore - recovered well from that. then diarrhoea which is slowly resolving. exam - 3/5 lame left fore. swollen distal to left elbow - antebrachium. no wounds found. tnder on palpation. no fluid build up. pyrexia - 39.7C. general exam otherwise NAD. injection convenia. see Friday for rads INI. possible bite/penetrating njury that started this and not fully resolved? Next appointment in 2 days”	[01] Certain infectious or parasitic diseases [13] Diseases of the digestive system [14] Diseases of the skin [15] Diseases of the musculoskeletal system or connective tissue
“DIARRHOEA. OR d++ last few days, since early yesterday morning, runny and bit mucousy but no blood seen. No recent change to diet or access to toxins but scavenges a lot. Otherwise usual self. O has starved her but not improved. Still good appetite and thirst. Neutered. Straining a little. BAR HR 124 strong and regular RR panting. eyes bright and clear, mm pink and moist, crt 1.5 secs, ears ok. abdo palp umcomfortable cranially and tensing up, ln’s mildly enlarged. skin and coat ok. normal hydration. T 38.6. Discussed possible GE viral/bacterial, pancreatitis, FB. Had a very nasty episode of HGE in January this year. Advised adntibiotics for colitis type signs and also probiotic, re-examine in 3 days or sooner if concerned. . Next appointment in 3 days”	[01] Certain infectious or parasitic diseases [13] Diseases of the digestive system
“last Tuesday wasn’t him self sittting and not knowing where to put himself, but then the next day perked up owner gave him a chewy bone, still has got plantigrade stance left hind, has signs of OA, Meloxaid helped but got d+ when on it, discussed Librela injection multiple lumps R/O lipomas, also top of head 5 mm × 5 mm sized skin growth R/O wartlike lesion, monitor, if doubles in size or bleeds further cours of action, both eyes nuclear sclerosis, oral cavity tar tar build up upper molars, Wt = 35.6 kg manipulation/palpation ojoints some stiffness, repeat librela in 1 month can be administered by nurse. Next appointment in 4 weeks.”	[02] Neoplasms [09] Diseases of the visual systems [14] Diseases of the skin [15] Diseases of the musculoskeletal system or connective tissue [23] Dental

To evaluate the utility of PetBERT as the base model for PetBERT-ICD in classifying veterinary EHR, existing BERT models with a thematic similarity developed using biomedical corpora were evaluated in the same classification tasks used for training and testing PetBERT-ICD. Models were trained using the same 250,000 training and evaluation sets created above, all models using the same mini-batch size of 32, initial learning rates of 5e−5, seeds, and were all trained with early stopping enabled, such that when evaluation loss increased, training stopped. The models that we compared with PetBERT were BioBERT^21^, ClinicalBERT^20^, and VetBERT^46^. We also included the original BERT-base model as a baseline for comparison. F1 score results are shown in Table 3 with the full precision and recall metrics accessible in the supplementary Table 1. As anticipated, BERT-base performed the poorest, achieving an overall F1 score micro-average of 0.81. This performance did not significantly improve when transitioning to BioBERT, with VetBERT and ClinicalBERT showing only a 1% improvement. PetBERT outperformed the other models, reporting the highest F1 score micro-average across most individual classes and achieving an overall improvement of 2%. We conducted a Friedman’s analysis of variance test to assess the statistical significance of the differences between BERT-Base and the examined models. Statistical significance was observed in the differences between BERT-Base and PetBERT ($\chi ^2$ = 14.45, *p* < 0.01). A comprehensive variance comparison is available as supplementary Tables 2 and 3.

**Table 3:** Performance comparison of fine-tuned BERT models: F1 scores on a 1000 EHR professionally annotated test set with relative differences compared to BERT-base. Highlighted in bold are the top-performing models within each ICD-11 syndromic disease coding class. Friedman variance test values ($\chi ^2$) and *p*-values compared to BERT-base.

	BERT-Base	BioBERT	ClinicalBERT	VetBERT	PetBERT-ICD
[01] Certain infectious or parasitic diseases	**0.78**	0.76 (− 0.02)	0.77 (− 0.01)	0.77 (− 0.01)	**0.78 (0.00)**
[02] Neoplasms	0.86	0.89 (+ 0.03)	0.88 (+ 0.02)	0.86 (0)	**0.92 (+ 0.06)**
[03] Diseases of the blood or blood-forming organs	0.50	0.67 (+ 0.17)	0.67 (+ 0.17)	**0.80 (+ 0.3)**	0.67 (+ 0.17)
[04] Diseases of the immune system	0.68	0.77 (+ 0.09)	0.79 (+ 0.11)	0.78 (+ 0.1)	**0.80 (+ 0.12)**
[05] Endocrine, nutritional or metabolic diseases	0.78	**0.79 (+ 0.01)**	0.76 (− 0.02)	0.78 (0.00)	0.78 (0.00)
[06] Mental, behavioural or neurodevelopmental disorders	0.77	0.74 (− 0.03)	0.77 (0)	**0.78 (+ 0.01)**	**0.78 (+ 0.01)**
[08] Diseases of the nervous system	0.60	**0.78 (+ 0.18)**	0.75 (+ 0.15)	0.71 (+ 0.11)	**0.78 (+ 0.18)**
[09] Diseases of the visual system	**0.89**	**0.89 (0.00)**	**0.89 (0.00)**	**0.89 (0.00)**	**0.89 (0.00)**
[10] Diseases of the ear or mastoid process	0.84	0.88 (+ 0.04)	0.88 (+ 0.04)	**0.89 (+ 0.05)**	**0.89 (+ 0.05)**
[11] Diseases of the circulatory system	0.79	**0.89 (+ 0.10)**	0.85 (+ 0.06)	**0.89 (+ 0.10)**	**0.89 (+ 0.1)**
[12] Diseases of the respiratory system	0.87	0.86 (− 0.01)	**0.89 (+ 0.02)**	0.88 (+ 0.01)	**0.89 (+ 0.02)**
[13] Diseases of the digestive system	0.84	0.89 (+ 0.05)	**0.91 (+ 0.07)**	0.87 (+ 0.03)	0.89 (+ 0.05)
[14] Diseases of the skin	0.76	0.75 (− 0.01)	**0.80 (+ 0.04)**	0.77 (+ 0.01)	0.79 (+ 0.03)
[15] Diseases of the musculoskeletal system or connective tissue	0.85	0.91 (+ 0.06)	0.91 (+ 0.06)	**0.93 (+ 0.08)**	**0.93 (+ 0.08)**
[16] Diseases of the genitourinary system	0.71	0.77 (+ 0.06)	0.80 (+ 0.09)	0.81 (+ 0.1)	**0.83 (+ 0.12)**
[18] Pregnancy, childbirth or the puerperium	0.89	0.95 (+ 0.06)	0.94 (+ 0.05)	0.94 (+ 0.05)	**1.00 (+ 0.11)**
[19] Certain conditions originating in the perinatal period	0.40	0.36 (− 0.04)	0.6 (+ 0.2)	0.6 (+ 0.2)	**0.67 (+ 0.27)**
[20] Developmental anomalies	0.18	0.11 (− 0.07)	**0.24 (+ 0.06)**	0.09 (− 0.09)	0.21 (+ 0.03)
[22] Injury, poisoning or certain other consequences of external causes	0.71	0.74 (+ 0.03)	0.69 (− 0.02)	0.73 (+ 0.02)	**0.80 (+ 0.09)**
Dental	0.83	0.83 (0)	0.86 (+ 0.03)	0.86 (+ 0.03)	**0.88 (+ 0.05)**
Micro average	0.81	0.81 (0)	0.82 (+ 0.01)	0.82 (+ 0.01)	**0.83 (+ 0.02)**
Macro average	0.77	0.76 (− 0.01)	0.78 (+ 0.01)	0.78 (+ 0.01)	**0.79 (+ 0.02)**
Weighted average	0.82	0.82 (0)	0.83 (+ 0.01)	0.83 (+ 0.01)	**0.84 (+ 0.02)**
Friedman test values ($\chi ^2$)	–	1.80	7.20	7.45	14.45
Friedman test p-values (*p*	–	1.18	$<0.01$	$<0.01$	$<0.01$

### Case study: disease outbreak analysis

For the direct comparison of the MPC system against the ICD-11 labelling framework, a previously identified disease outbreak between December 2019 and March 2020 recognised in the SAVSNET data (canine enteric coronavirus) was used as a source of reference to validate if such an outbreak could be observed using PetBERT-ICD. Given the principle clinical presentation of vomiting, this was aligned with the ‘gastroenteric’ MPC and the ‘Diseases of the Digestive System’ ICD-11 label. The increase in frequency was reflected in an increased proportion of consultation records labelled with the ‘gastroenteric’ MPC. The authors hypothesised that records labelled with the *‘Diseases of the digestive system’* ICD-11 label would similarly detect the outbreak. Given that the aetiological agent in this outbreak was an infectious agent(canine enteric coronavirus)^38^, a further test of pairing the *‘Diseases of the digestive system’* and *‘certain infectious and parasitic diseases’* ICD-11 labels together was also considered.

The binary response variable for each of the three above conditions is represented as $Y_{j,i,t}$ if a given consult *j* at a given location *i* on a given time *t* is one if the system identifies gastrointestinal consults and zero for anything else. Daily counts were taken from the test set between 29th February 2014 and 1st October 2022, comprising a total of 1,431,506 canine EHRs, of which 50,140 were annotated with a gastroenteric MPC system 147,451 for the single label *‘Diseases of the digestive system’* and 45,311 for the dual label *‘Diseases of the digestive system’* and *‘Certain Infectious and Parasitic Diseases’*, with all records not labelled with the target labels used as the denominator. The data was only analysed for records from dogs, as this was the species affected by the outbreak. A spatiotemporal mixed effects regression model was fitted using Bayesian inference. The model has two components: the fixed effect using multiple linear regression and the random effect to outline a latent stochastic process to account for unexplained fluctuations within a large population across space and time. These are considered by a latent spatially and temporally correlated stochastic process defined as $S_{i,t}$ at location *i* and time *t* with an expected value close to zero. A user-inputted threshold *l* is defined as a reference to determine when $S_{i,t}$ increases above the typical, expected value. Where the predictive probability *q*, calculated from all available data within *t* for each location and time, exceeds *l* an outbreak at a given location is declared. For the predictive probability, *M* posterior samples ${{S}_{i,t}^{(1)},\ldots ,{S}_{i,t}^{(M)}}$ were generated from the joint predictive distribution of the random effects $S_{i,t}$ using a Markov Chain Monte Carlo algorithm. Sufficient convergence required a burn-in period of 5k iterations with a further *M*= 50k iterations. A more complete outline of the method, including the sudden fluctuation in attending veterinary consultations during the COVID-19 pandemic, has been described elsewhere^47,48^.

$$\begin{aligned} {q}_{i,t}=\frac{1}{M}\mathop {\sum }\limits _{m=1}^{M}\mathrm{{I}}({S}_{i,t}^{(m)} > l) \end{aligned}$$

**Figure 2:**
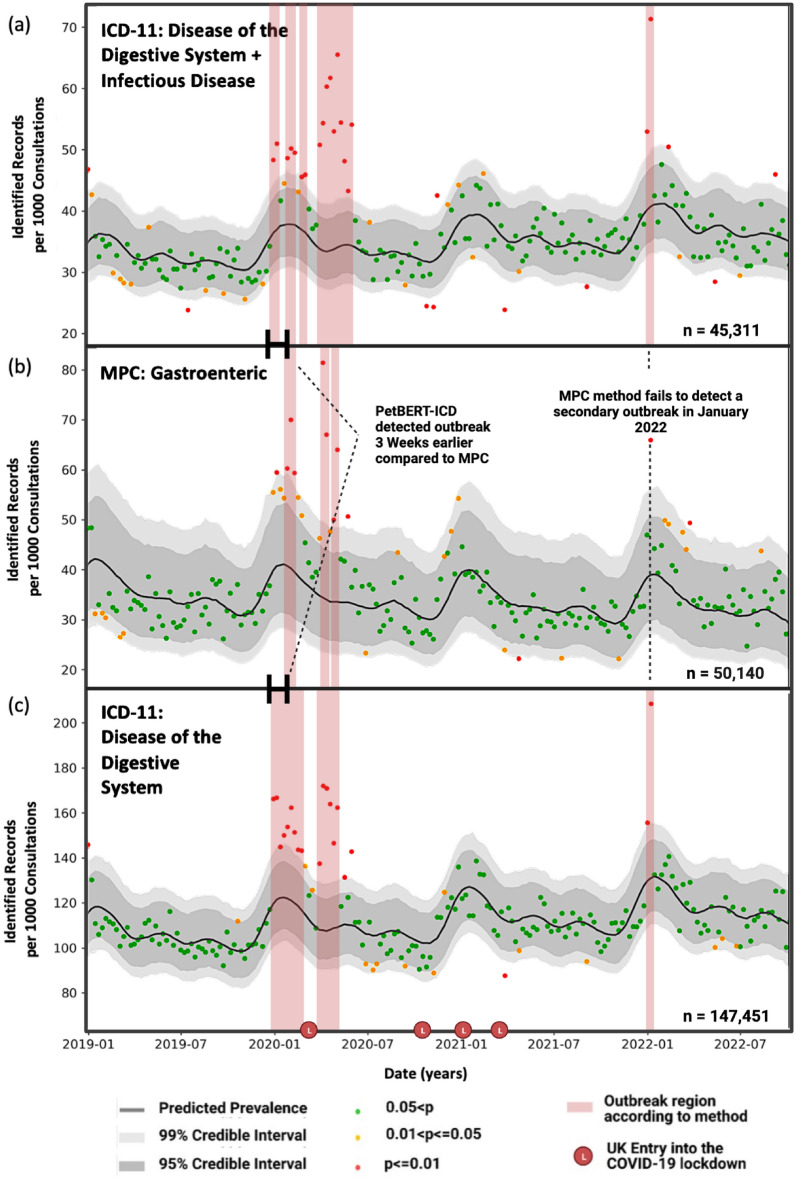
Prevalence of canine records observed through three frameworks: (**a**) ICD-11 paired ‘diseases of the digestive system’ and ‘certain infectious and parasitic diseases,’ (**b**) MPC clinician-assigned gastroenteric label, and (**c**) ICD-11 ‘diseases of the digestive system only.’ Each point represents a 1-week period. The regression line indicates estimated prevalence with 95% and 99% credibility intervals. Areas in red represent potential outbreak periods. ‘L’ indicators on the bottom axis mark the onset of COVID-19 lockdowns in the UK.

All three strategies successfully captured the 2019 canine enteric outbreak (Fig. 2). Each point represents a week, where when it exists within the 95% credible interval, it is suggestive that a normal period is occurring. Fluctuations within the predicted prevalence, particularly during the winter periods, are representative of the general seasonality of gastrointestinal diseases within the UK^49^. The UK’s entry into the COVID-19 lockdown also resulted in changes in consultation patterns, with significantly fewer presenting cases, and those who did were likely more serious; there was an increase in telephone consultations during this time, also decreasing the already limited diagnostic capabilities of clinicians further^50,51^. The COVID-19 impact was considered within the modelling process; however, the dramatic variance in the number of consultations may have impacted the predicted prevalence. Points exceeding this range, such as those beyond the 99% credible intervals for two consecutive weeks, are considered a potential outbreak. A more significant clustering of values exceeding these credible intervals was identified during the first outbreak period, satisfying the outbreak definition. With the dual labelling ICD-11 methods, this outbreak was realised a full 3 weeks before the MPC system, and the single ICD-11 label also 2 weeks before. A second outbreak in January 2022 was suggested by the ICD-11 data using the single Diseases of the Digestive System and paired label strategies. However, it was completely absent in the MPC labelling strategy.

## Discussion

First-opinion clinical notes lack diagnostic certainties. This situation is intrinsically linked to the inherent private healthcare infrastructure within the UK’s veterinary landscape. Financial considerations frequently prompt pet owners to seek alternatives to diagnostic tests and specialist referrals, opting instead to rely on advice from their primary care practitioners. Additional factors, including the level of client care for animals and client education, further shape this landscape. Consequently, first-opinion practitioners often lack (or do not need to apply) the definitive tools for confirming diagnoses, leading to listing potential differential diagnoses and selecting treatments based on informed estimates. In most clinical narratives, a specific diagnosis is often omitted from clinical narratives but replaced by discussions of various differentials. This entails a degree of vagueness for many of the EHRs within the dataset, where a presenting symptom can often be explanatory to many different conditions and frequently contradictory. Adding to this complexity is the lack of data conventions within the free-text inputs and the inconsistencies between clinicians regarding spellings and abbreviations, resulting in further ambiguity in understanding the consultation events.

Here, we introduce the first opinion veterinary-trained language model, ‘PetBERT’, trained on over 500 million tokens from veterinary practices across the UK. We have applied PetBERT to the downstream annotation task of high-level ICD-11 disease coding. To achieve this, we used a semi-supervised two-stage process that incorporated individual binary classification models applied to a single dataset, which was then used to train the multi-label classification model PetBERT-ICD. This yielded an average F1-score across our labels exceeding 83% and will now be continually applied to our dataset for monitoring the disease and syndromes of the companion animal population in the UK. PetBERT achieved the highest F1 score across 15 of the 20 syndromic codings (75%) and outperformed BERT-base in 17 of the 20 codings (85%). This represents an overall average improvement of 2%, suggesting that the additional pretraining steps using the masked language approach were productive. Of the three instances where no improvement over BERT-base was observed, this additional pretraining had no adverse consequence. The exact reasoning for no improvement is unclear but may suggest linguistic terms not differing drastically from the non-clinical language that BERT-base was trained on. While the known outbreaks in this study yielded stronger disease signals, smaller outbreaks may require the increased sensitivity of PetBERT. Implementing methodologies that enhance the precision of disease capture can lower the threshold for detecting disease occurrence anomalies, consequently increasing the rate of detection. The method to divide the dataset by institutions provides robust confidence in the generalisability of the model, with new practices or clinicians contributing to the dataset in the future should not be expected to significantly change the model’s performance. With fewer than 23% of healthcare ML research being from multi-institutional datasets, it is essential to consider what unintended biases are inherently being attained through training and validating on a single dataset, with several studies showing that models considered useful deteriorate significantly when applied to out-of-institution data^52–54^.

The semi-supervised teacher-student modelling approach across the ICD-11 codings performed significantly better than the MPC labels attached by the veterinary practitioners at point-of-care. Disease coding is a challenging task prone to errors, exacerbated by the time and workload constraints placed on practitioners. Other works have shown that disease coding annotations are vulnerable to high error rates, with some recording as high as 90%^44,45^. For the annotations of the ICD-11 dataset, there were more labels to select from, and there was no restriction to a single label, rather, any label that fitted the case definition was applied, removing a significant degree of ambiguity that clinicians may face when applying MPCs. The SAVSNET MPC labelling system takes a more reductionist approach than the ICD-11 system; practitioners can only assign one label with fewer options. This decision was made in light of minimising clinician time when submitting a record to SAVSNET. Annotations for the ICD-11 labels were not done in the stressful clinical environment, allowing the privilege of annotating records without time and pressure constraints , thus, improving accuracy. Records annotated by clinicians among their duties are particularly prone to incomplete data and inputting data long after procedures have occurred, leading to errors being made^55^. The two worst performing labels within the MPCs are the “other healthy” and “other unwell” which broadly were for conditions that did not fit appropriately into any other category or records that were incorrectly annotated. These labels comprise over 44% of the entire SAVSNET dataset. Of the 1000 professionally annotated records, some 435 records were assigned by the attending clinician at the point of consultation with “other healthy” or “other unwell”. Over 61% of these records could be reclassified into one of the eight disease-encoding MPCs. Given the error rate in MPC annotation by attending clinicians, it is clear that the accuracy of these annotations must be carefully assessed. Therefore, it should be considered whether it is appropriate to request codings from clinicians with such high error rates, which is particularly relevant for the scenario presented here where, ultimately, the final form of disease classification will not consider the annotations made by the clinicians. As more medical datasets will be used in training machine learning models, these inevitable flaws in annotation errors made by clinicians will intrinsically influence the models^56,57^. Machine learning models that inherit the same human errors within their training sets will ultimately reproduce the same errors as the clinicians themselves.

The canine enteric coronavirus outbreak of December 2019 to March 2020 was identified by Radford et al. 2021^38,48^ and was detected through changes in the frequency of the ‘gastroenteric’ MPC veterinarian-derived labels (as well as changes in prescribing for anti-emetic drugs). Although this has proved a valid methodology in realising outbreaks, it is not without flaws in the aforementioned labelling errors that clinicians may assign reducing disease signals. Although the clinical signs characterising the outbreak (vomiting, diarrhoea, poor appetite) were covered within one of the available MPC classes, the MPC framework might not generate such clear disease signals from future outbreaks if clinical signs could be attributed to multiple MPC classes or to syndromes not covered by specific MPCs. One example is the canine distemper outbreak in Bari, Italy, in March 2003^58^. The main presenting symptoms were fever, conjunctivitis, respiratory distress and enteritis. Using the MPC framework, vets could equally select ‘ocular’, ‘respiratory’, ‘gastroenteric’ or ‘other$\_$unwell’, potentially diluting a signal for each outbreak detection capability. The advantage of a multi-label classifier is that it enables the assignment of several labels together. This allows for an increase in all selected labels and for the possibility of considering diseases as triggering a cluster of labels. This idea was examined here by considering the *‘Diseases of the digestive system’* and the *‘Certain infectious and parasitic diseases’* in combination. PetBERT-ICD is a better tool for disease outbreak detection, identifying an outbreak (signal greater than 99% credibility limit for two consecutive weeks) up to 3 weeks earlier than the MPC method for the first outbreak and potentially detecting the second outbreak of 2022, which the MPC framework failed to identify. These findings highlight the potential of PetBERT-ICD as a valuable tool for timely and effective disease outbreak detection. During an outbreak, having a time advantage lead can be critical to providing helpful public advice, minimising the number of infections, reducing the epidemic potential, and ultimately safeguarding public health. The outbreak is represented across multiple periods instead of a continuous event; we can speculate that the model represents different waves of the epidemic disease, attributed mainly to the interplay of biology and behavioural factors. Alternatively, the UK’s entry into the COVID-19 lockdown impacted the number of cases presented to a clinician and the minimising of social contact.

Although every effort was made to verify that the records assigned to a given ICD-11 class were correct, some records may inevitably have been missed or incorrectly labelled. The choice to use ICD-11 highest order labels was selected based on the challenges of converting many human-related conditions to the context of veterinary care and the additional complexity this would have placed on our annotators. Future works may supply an increased resolution of classification by focusing on individual branches of the ICD-11 hierarchy and, therefore, be able to more closely track individual conditions rather than the syndromic approach presented here. Furthermore, we encountered challenges in finding underrepresented syndromes, such as *‘Diseases of the blood or blood-forming organs’*. Although our test methodologies were sufficient in verifying our model’s performance metrics, increasing the frequency of these syndromes within our training and test sets would have reduced the degrees of variance and uncertainties observed. The modular approach of creating individual binary classifiers for each disease coding offers flexibility for improving the performance of the PetBERT-ICD classifier in the future. Moreover, this pipeline can readily adapt to future model architectures, potentially enhancing performance. The model’s results represent the current state of language clinicians use as of the time of writing. Whilst the transformer architecture and decision to split datasets on unseen clinician notes evoke confidence in the generalisability of PetBERT performance metrics, evaluating the quality of the generated annotations must be continually monitored. In the current study, individual consultation records were considered independent events. Consequently, any mentions of chronic or ongoing conditions in previous consultations, unless mentioned again by the attending clinician, will not be carried forward and influence the current annotation. The animals within this study are limited by those attending practices associated with SAVSNET, with the contents of the free-text consultation notes entirely dependent on the attending clinician.

To conclude, PetBERT is a masked language model based on the BERT architecture further trained on over 500 million additional words from first-opinion veterinary clinicians from across the UK. This study has demonstrated the capability of PetBERT-ICD in the automated ICD-11 multi-label classification tool for first-opinion veterinary free-text EHRs, achieving an F1 score exceeding 83%, providing a novel method for the mass surveillance of the UK companion animal population. We highlight the challenges associated with point-of-care annotations and why manual annotations outside such a setting yield much more robust results. Our two-step strategy to develop individual binary classification models onto a singular dataset for multi-label training maximised the performance of PetBERT-ICD despite the small number of human annotations. Finally, we demonstrate PetBERT-ICD’s utility in identifying disease outbreaks within the small animal companion population through the example of the previous canine enteric coronavirus outbreak. Our new method captures much broader syndromes and can apply distinct combinations to form a higher-resolution scope of the outbreak events. The ability of PetBERT-ICD to detect outbreaks up to 3 weeks earlier than current clinician-assigned point-of-care labelling strategies could mean the difference between a localised outbreak and a more widespread epidemic. The method also provides a smaller credible interval range, suggesting more robust, higher precision values. This system will now be continually applied to all future records for the detection of future outbreaks.

## Methods

### Datasets

Electronic health records have been collected since March 2014 by SAVSNET, the Small Animal Veterinary Surveillance Network, comprising a sentinel network of 253 volunteer veterinary practices across the United Kingdom. A complete description of SAVSNET has been presented elsewhere^5^. In summary, based on convenience, veterinary practices with compatible practice management software with the SAVSNET data exchange are recruited. Within these participating practices, data is collected from each booked consultation (where an appointment has been made to see a veterinary practitioner or nurse). All owners within these practices can opt-out at the time of consultation, and therefore, their data will be excluded. Data is collected on a consultation-by-consultation basis and includes information such as species, breed, sex, neuter status, age, owner’s postcode, insurance and microchipping status, and crucial to this study, a free-text clinical narrative outlining the events that occurred within that consultation. At the end of each consultation, veterinary practitioners are given 10 ‘main presenting complaint’ (MPC) groups to categorise the main reason the animal presented; these are gastrointestinal, respiratory, pruritus, tumour, renal, trauma, post-operative checkups, vaccination and, other healthy and other unwell. Sensitive information, such as personal identifiers, is cleaned from the data without further preprocessing. SAVSNET has ethical approval from the University of Liverpool Research Ethics Committee (RETH001081). Table 4 summarises the cleaned SAVSNET dataset after the above process for cats and dogs only. We segregated EHRs into training and testing sets based on their respective source practices. This stratification approach ensures that the clinical notes used for testing were separate from those generated by clinicians who had contributed to the training sets, thereby fortifying the robustness of our results and mitigating potential bias.

**Table 4:** SAVSNET dataset overview of the high-level variables of the companion animal distribution of cats and dogs by species, sex, age, neuter status, and the selected MPC between March 2014 to November 2022.

Variable	Level	Dogs	Cats
Species	Dogs	5,275,843	–
	Cats	–	2,062,074
Sex	Male	2,710,641 (51.2%)	1,009,388 (48.1%)
	Female	2,565,202 (48.8%)	1,052,686 (51.9%)
Country	England	4,7152,76 (90.4%)	1,871,536 (91.6%)
	Scotland	252,024 (4.8%)	81,883 (4.2%)
	Wales	216,799 (4.2%)	77,774 (3.9%)
	Northern Ireland	34,129 (0.6%)	6204 (0.3%)
Age	Infant (0 to 1 year)	501,339 (11.9%)	190,534 (9.8%)
	Adult (1–10 years)	2,830,739 (64.5%)	887,640 (51.0%)
	Senior (10 years)	1,036,075 (23.6%)	691,738 (39.2%)
Neutered	Yes	3,587,028 (68.0%)	1,670,280 (81.4%)
	No	1,688,093 (32.0%)	391,794 (19.6%)
MPC	Gastroenteric	174,688 (3.3%)	45,368 (2.4%)
	Kidney$\_$disease	14,046 (0.2%)	18,169 (0.9%)
	Other$\_$healthy	1,333,760 (25.4%)	494,170 (23.7%)
	Other$\_$unwell	1,006,031 (19.6%)	418,107 (21.3%)
	Post$\_$op	414,764 (7.8%)	135,865 (6.6%)
	Pruritus	283,880 (5.3%)	53,869 (2.7%)
	Respiratory	52,625 (0.9%)	27,123 (1.3%)
	Trauma	249,039 (4.7%)	102,646 (4.9%)
	Tumour	100,080 (1.8%)	23,865 (1.1%)
	Vaccination	1,639,268 (31.0%)	739,890 (35.1%)

For PetBERT-ICD Multi-label classifier training, 7500 random EHRs were extracted from the training dataset; these records were annotated against 20 high-level ICD-11 labels. As some labels are rarely present within this random sampling method, common words associated were used as search terms to increase the probable likelihood of records appearing, for example, to reveal more records associated with ‘Pregnancy, childbirth or the puerperium’, search words such as “pregnancy, pregnant, labour, birth” were used. Records were annotated, ranging from no label with the capability for all 20, with multi-label annotations for a single narrative also being permitted. The 20 labels selected are the ICD-11 highest level codings, disregarding the ‘Sleep-wake disorders’ and ‘Conditions related to sexual health’ due to limited disease presence within veterinary EHR. Additionally, a label to identify dental conditions was differentiated from the *‘Diseases of the digestive system’* as stated within the ICD-11 framework; the large volume of dental conditions within the dataset warranted its own label. Table 1 shows the total in-class records for each of the 20 datasets.

### Model architecture and training

#### Additional pretraining of PetBERT

Adaption of the ULMFiT framework was utilised in the production of ‘PetBERT’ based upon minimal modifications to the BERT architecture^19^. Firstly, the pre-trained BERT-base model previously exposed to the general-purpose language of Wikipedia and BooksCorpus was further fine-tuned on the 500 million token dataset of first opinion clinical free-text narratives on a simultaneous training task of Masked Language (MLM) and Next Sentence Prediction (NSP), mimicking the tasks used in the initial pre-training of BERT^12^. For MLM training, 15% of the words within a given clinical narrative were masked randomly across the entire training dataset. The model was tasked to substitute the masked word with a suitable word, requiring a deep bidirectional understanding of the text. For NSP training, sentences between narratives were randomly split and rejoined either to the same sentence or to a random sentence with a [SEP] token in between. The model had to determine whether the new sentence pairs made sense, enabling a cross-sentence understanding of the text. The model had a 10% evaluation set created randomly to calculate a validation loss to determine the number of training epochs required. The training ended when evaluation loss increased, occurring beyond epoch 8, with the final model selected for downstream tasks. Training took 450 hours on a single Nvidia A100 GPU.

#### Fine tuning PetBERT for ICD-11 classification PetBERT-ICD

To create the training set for the multi-label ICD 11 classifier, individual binary sequence classification models for each of the 20 target labels were produced. A semi-supervised Teacher-Student model approach, adapting the works of Yalniz et al. 2019, was utilised where a small number of manually annotated records containing the given class and controls in equal proportions was used to train a small PetBERT-based binary sequence classification model^42^. This approach efficiently uses a limited number of manually annotated records and optimises multiple smaller models on more straightforward tasks. Due to their relatively small training sizes, as outlined in Table 1, these models were fast to train, often taking less than an hour for each with a given mini batch size of 16, an initial learning rate of 2e−5 with early stopping enabled to determine the optimal number of epochs, with the average at epoch six. This was an iterative process whereby the results from the given model were applied to a small sample of unseen records to verify model performance. Where necessary, training sets were modified to reflect model mistakes. Out-of-class EHRs were shared among the binary sequence classification models such that the models could more distinctly identify differences despite often sharing similar word semantics. For example, the positive cases for *‘Diseases of the Respiratory’* were used as the negative controls for *‘Diseases of the Circulatory’* and vice versa. This was also performed between *‘Endocrine, nutritional and metabolic diseases’* and *‘Diseases of the digestive system’*, *‘Diseases of the skin’* and *‘Diseases of the ear or mastoid process’*, and *‘Pregnancy, childbirth or the puerperium’*, *‘Developmental anomalies’*, and *‘Certain conditions originating in the perinatal period’*.

**Figure 3:**
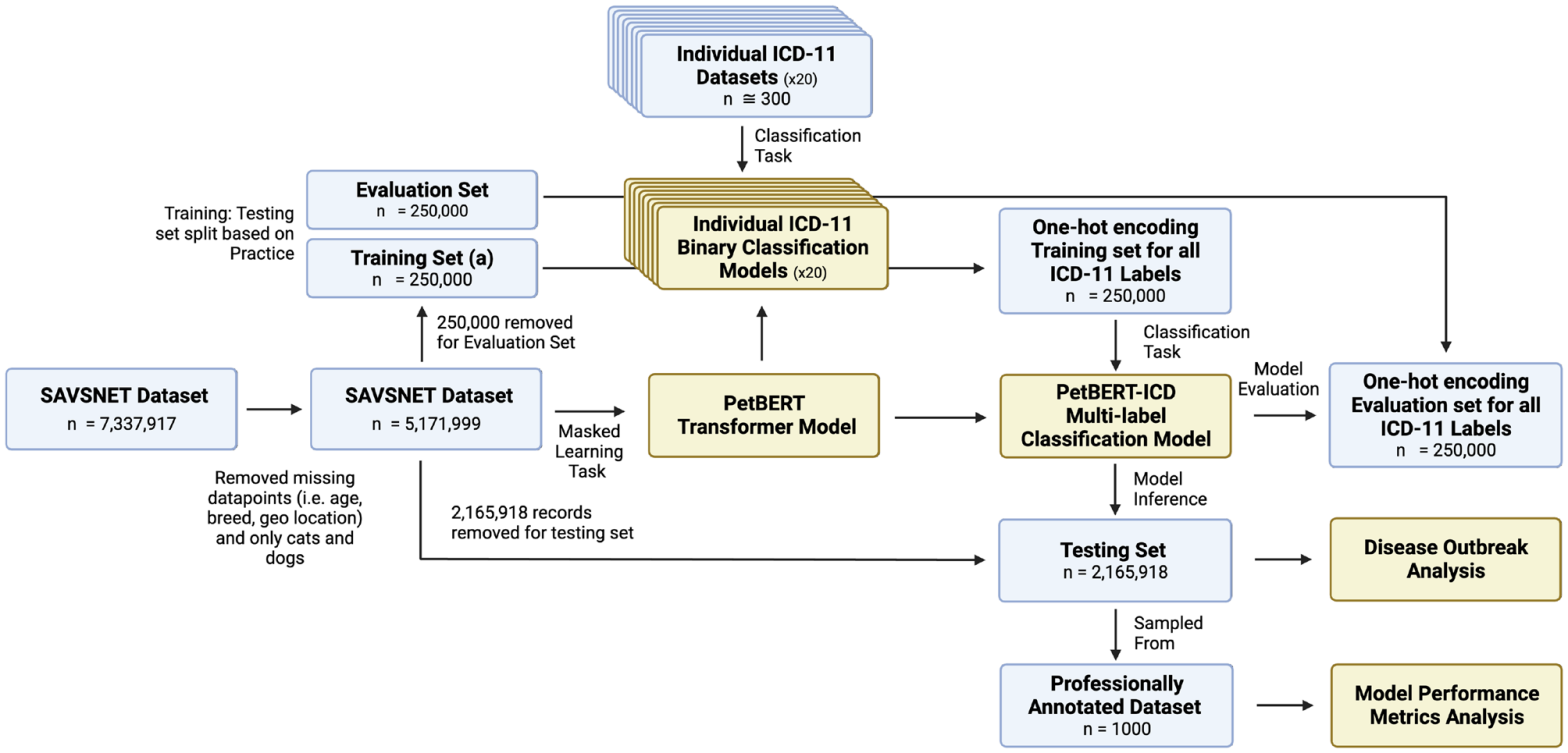
Data pipeline for the creation of the ensemble multi-label ICD-11 classification model, PetBERT-ICD, derived from the 20 binary classification models, with final application onto the 2.1 million unseen records for the outbreak detection analysis. From this 2.1 million, a random 1000 records were sampled and were annotated by a practising clinician to evaluate model performance. (n = number of samples).

The resultant 20 binary classification models were then individually applied against the 250k “training set (a)” dataset and the 250k “Evaluation set”; each EHR could have up to 20 labels represented as a one-hot encoding vector. The Multi-label classification model containing the 20 labels was then trained based on this newly derived dataset with a weighted binary cross-entropy loss function with sigmoid activation to account for differences in class sizes. The training continued until evaluation loss began to increase, occurring beyond epoch 8, with a mini-batch size 32 and an initial learning rate of 2e−5 using the AdamW optimiser. Training for the multi-label classifier took approximately 75 hours on a single Nvidia A100 GPU. We performed an iterative threshold analysis on the evaluation set to determine the optimal threshold for PetBERT-ICD to assign labels. This process included assigning labels at 5% intervals within the range of 60% to 95%, and subsequently assessing the corresponding recall scores. Recall was prioritised over precision to minimise the occurrence of false negatives in disease detection. Our investigation revealed that the most effective threshold for optimal recall was identified at 80%. The ICD-11 classifier was then applied against the 2.1 million testing set. Figure 3 shows a complete outline of the training process.

### Model evaluation

To validate our methodology for training 20 binary classification models, which are used to generate a multi-label dataset for training PetBERT-ICD, we assessed the performance of the final PetBERT-ICD model on an additional evaluation set created using the same 20 binary classification models. A high degree of agreement between the multi-label classifier and the 20 binary classifiers would indicate that the multi-label classifier maintains its performance effectively, with minimal to no reduction in performance compared to the individual binary classifiers. Subsequently, PetBERT-ICD was applied to the complete testing set of 2.1 million records, of which 1000 records were taken from this sample to be given to the attending clinician for final annotations and model metrics to be performed. From these 2.1 million records, we performed an additional case study, focussing on disease outbreak analysis to assess PetBERT-ICD’s performance against the MPC clinician-derived labelling system.

To assess and compare PetBERT’s performance against other models that were thematically aligned with biomedical corpora, we trained a group of models using the same 250,000 training sets for multi-label classification. To ensure equitable comparison, a standardised training protocol was employed, where all models adhered to an identical mini-batch size, learning rate, seed, and early stopping criteria mirroring the parameters used for training PetBERT. We applied all models to the same test set and measured their relative performances. The models we selected for the comparison were BioBERT^21^, ClinicalBERT^20^, and VetBERT^46^. BioBERT is a BERT model that was further trained on 18 billion words from PubMed and PMC texts^21^. ClinicalBERT is a BioBERT model that was further trained on 0.5 billion words from the MIMIC-III corpus^20,59^. VetBERT is a ClinicalBERT model that was further trained on 15 million clinical notes from VetCompass Australia corpus^46^. As a baseline, the original BERT-base model^12^ was also incorporated into our analysis to determine the relative significance of disparities in outcomes among the models. We employed a Friedman Test to aid in discerning meaningful differences across each model’s performance.

$$\begin{aligned} \chi ^2 = \frac{12}{nk(n+1)} \sum _{j=1}^{k} R_j^2 - 3n(k+1) \end{aligned}$$

   Where $\chi ^2$ is the Friedman test statistic, *n* is the number of observations, *k* is the number of classifiers, and $R_j$ is the sum of ranks for each classifier *j*. Individual Friedman scores were produced for each classifier relative to BERT-base. Therefore, significance was determined via the *p*-value being lower than the $\alpha$ (0.05). This approach provided us with a robust means of assessing the relative performance of PetBERT compared to these state-of-the-art models, offering increased confidence in our findings.

### Supplementary Information

Supplementary Tables.

## Data Availability

The datasets analysed during the current study are not publicly available due to issues surrounding owner confidentiality. Reasonable requests can be made to the SAVSNET Data Access and Publication Panel (savsnet@liverpool.ac.uk) for researchers who meet the criteria for access to confidential data.

## Code availability

The source code for the data preprocessing, dataset splitting and training of both PetBERT and PetBERT-ICD is publicly available on GitHub at the following repository: https://github.com/SAVSNET/PetBERT.
