# Supplementary material for: PetBERT: automated ICD-11 syndromic disease coding for outbreak detection in first opinion veterinary electronic health records
Source: Sci Rep. 2023 Oct 21;13:18015. doi: 10.1038/s41598-023-45155-7 (PMC10590382; doi:10.1038/s41598-023-45155-7)
Supplement: Supplementary file 1 — Supplementary Tables. [file 41598_2023_45155_MOESM1_ESM.pdf]

Table 1. Comparison of similar thematic fine-tuned BERT models precision and recall metrics on the 1000 EHR professionally annotated test set. Relative difference is compared to the base model for all other models as BERT-base

	BERT		BioBERT		BioClinical		VetBERT		PetBERT	
	**Precision**	**Recall**	**Precision**	**Recall**	**Precision**	**Recall**	**Precision**	**Recall**	**Precision**	**Recall**
**[01] Certain infectious or parasitic diseases**	0.68	0.90	0.66 (-0.02)	0.93 (0.03)	0.66 (-0.02)	0.92 (0.02)	0.69 (0.01)	0.86 (-0.04)	0.69 (0.01)	0.88(-0.02)
**[02] Neoplasms**	0.93	0.86	0.93 (0.00)	0.88 (0.02)	0.88 (-0.05)	0.87 (0.01)	0.81 (-0.12)	0.92 (0.06)	0.91 (-0.02)	0.92 (0.06)
**[03] Diseases of the blood or blood-forming organs**	0.45	0.83	0.56 (0.11)	0.83 (0.00)	0.50 (0.05)	1.00 (0.17)	1.00 (0.55)	0.67 (-0.16)	0.50 (0.05)	0.50 (-0.33)
**[04] Diseases of the immune system**	0.80	0.82	0.70 (-0.1)	0.82 (0.00)	0.76 (-0.04)	0.82 (0.00)	0.76 (-0.04)	0.80 (-0.02)	0.76 (-0.04)	0.85 (0.03)
**[05] Endocrine, nutritional or metabolic diseases**	0.69	0.85	0.69 (0.00)	0.83 (-0.02)	0.68 (-0.01)	0.85 (0.00)	0.72 (0.03)	0.85 (0.00)	0.64 (-0.05)	0.85 (0.00)
**[06] Mental, behavioural or neurodevelopmental disorders**	0.70	0.84	0.67 (-0.03)	0.86 (0.02)	0.72 (0.02)	0.83 (-0.01)	0.73 (0.03)	0.83 (-0.01)	0.65 (-0.05)	0.91 (0.07)
**[08] Diseases of the nervous system**	0.86	0.62	0.87 (0.01)	0.69 (0.07)	0.86 (0.00)	0.66 (0.04)	0.82 (-0.04)	0.62 (0.00)	0.83 (-0.03)	0.77 (0.15)
**[09] Diseases of the visual system**	0.82	0.99	0.79 (-0.03)	1.00 (0.01)	0.80 (-0.02)	1.00 (0.01)	0.80 (-0.02)	1.00 (0.01)	0.81 (-0.01)	1.00 (0.01)
**[10] Diseases of the ear or mastoid process**	0.95	0.8	0.88 (-0.07)	0.85 (0.05)	0.94 (-0.01)	0.83 (0.03)	0.89 (-0.06)	0.88 (0.08)	0.89 (-0.06)	0.85 (0.05)
**[11] Diseases of the circulatory system**	0.97	0.78	0.97 (0)	0.83 (0.05)	0.94 (-0.03)	0.78 (0.00)	1.00 (0.03)	0.80 (0.02)	0.94 (-0.03)	0.85 (0.07)
**[12] Diseases of the respiratory system**	0.83	0.91	0.82 (-0.01)	0.93 (0.02)	0.85 (0.02)	0.93 (0.02)	0.85 (0.02)	0.91 (0.00)	0.82 (-0.01)	0.93 (0.02)
**[13] Diseases of the digestive system**	0.87	0.9	0.84 (-0.03)	0.91 (0.01)	0.92 (0.05)	0.91 (0.01)	0.86 (-0.01)	0.88 (-0.03)	0.86 (-0.01)	0.92 (0.02)
**[14] Diseases of the skin**	0.87	0.7	0.85 (-0.02)	0.73 (0.03)	0.86 (-0.01)	0.74 (0.04)	0.87 (0.00)	0.7 (0.00)	0.87 (0.00)	0.71 (0.01)
**[15] Diseases of the musculoskeletal system or connective tissue**	0.97	0.93	0.9 (-0.07)	0.94 (0.01)	0.91 (-0.06)	0.92 (-0.01)	0.91 (-0.06)	0.96 (0.03)	0.92 (-0.05)	0.93 (0.00)
**[16] Diseases of the genitourinary system**	0.79	0.81	0.7 (-0.09)	0.83 (0.02)	0.72 (-0.07)	0.89 (0.08)	0.80 (0.01)	0.83 (0.02)	0.75 (-0.04)	0.94 (0.13)
**[18] Pregnancy, childbirth or the puerperium**	1.00	0.89	0.90 (-0.10)	1.00 (0.11)	1.00 (0.00)	0.89 (0.00)	1.00 (0.00)	0.89 (0.00)	1.00 (0.00)	1.00 (0.11)
**[19] Certain conditions originating in the perinatal period**	0.60	0.50	0.60 (0.00)	0.50 (0.00)	0.75 (0.15)	0.50 (0.00)	0.75(0.15)	0.5 (0.00)	0.67 (0.07)	0.67 (0.17)
**[20] Developmental anomalies**	0.10	0.67	0.04 (-0.06)	0.33 (-0.34)	0.14 (0.04)	1.00 (0.33)	0.05 (-0.05)	0.33 (-0.34)	0.13 (0.03)	0.67 (0.00)
**[22] Injury, poisoning or certain other consequences of external causes**	0.58	0.87	0.51 (-0.07)	0.88 (0.01)	0.56 (-0.02)	0.9 (0.03)	0.64 (0.06)	0.84 (-0.03)	0.75 (0.17)	0.87 (0.00)
**Dental**	0.81	0.95	0.78 (-0.03)	0.91 (-0.04)	0.79 (-0.02)	0.93 (-0.02)	0.81 (0.00)	0.92 (-0.03)	0.77 (-0.04)	0.96 (0.01)

Table 2. Friedman Tests X^2r scores from individual label f1-score results

	BERT	BioBERT	ClinicalBERT	VetBERT	PetBERT
**BERT**	-	1.80	7.20	8.45	14.45
**BioBERT**	-	-	0.80	0.80	8.45
**ClinicalBERT**	-	-	-	0.05	6.05
**VetBERT**	-	-	-	-	7.21
**PetBERT**	-	-	-	-	-

Table 3. Friedman Tests p-values from individual label f1-score results

	BERT	BioBERT	ClinicalBERT	VetBERT	PetBERT
**BERT**	-	1.17971	0.00729	0.00365	0.00014
**BioBERT**	-	-	0.37109	0.37109	0.00365
**ClinicalBERT**	-	-	-	0.82306	0.01391
**VetBERT**	-	-	-	-	0.00729
**PetBERT**	-	-	-	-	-
